# Indentation Stiffness Measurement by an Optical Coherence Tomography-Based Air-Jet Indentation System Can Reflect Type I Collagen Abundance and Organisation in Diabetic Wounds

**DOI:** 10.3389/fbioe.2021.648453

**Published:** 2021-03-04

**Authors:** Harry Ming Chun Choi, Alex Kwok-Kuen Cheung, Michelle Chun Har Ng, Yongping Zheng, Yih-Kuen Jan, Gladys Lai Ying Cheing

**Affiliations:** ^1^Department of Rehabilitation Sciences, The Hong Kong Polytechnic University, Kowloon, Hong Kong; ^2^Department of Biomedical Engineering, The Hong Kong Polytechnic University, Kowloon, Hong Kong; ^3^Department of Kinesiology and Community Health, University of Illinois at Urbana-Champaign, Urbana, IL, United States

**Keywords:** biomechanical properties, collagen, diabetic wounds, non-invasive measurement, diagnostic device

## Abstract

There is a lack of quantitative and non-invasive clinical biomechanical assessment tools for diabetic foot ulcers. Our previous study reported that the indentation stiffness measured by an optical coherence tomography-based air-jet indentation system in a non-contact and non-invasive manner may reflect the tensile properties of diabetic wounds. As the tensile properties are known to be contributed by type I collagen, this study was aimed to establish the correlations between the indentation stiffness, and type I collagen abundance and organisation, in order to further justify and characterise the *in vivo* indentation stiffness measurement in diabetic wounds. In a male streptozotocin-induced diabetic rat model, indentation stiffness, and type I collagen abundance and organisation of excisional wounds were quantified and examined using the optical coherence tomography-based air-jet indentation system and picrosirius red polarised light microscopy, respectively, on post-wounding days 3, 5, 7, 10, 14, and 21. The results showed significant negative correlations between indentation stiffness at the wound centre, and the collagen abundance and organisation. The correlations between the indentation stiffness, as well as collagen abundance and organisation of diabetic wounds suggest that the optical coherence tomography-based air-jet indentation system can potentially be used to quantitatively and non-invasively monitor diabetic wound healing in clinical settings, clinical research or preclinical research.

## Introduction

Diabetes mellitus is a metabolic disease characterised by hyperglycaemia. Peripheral polyneuropathy, regional ischaemia in the limbs and foot ulceration are common diabetes-related complications. Due to impaired sensation and circulation, people with diabetes are susceptible to repeated cutaneous injuries and possible wound infection, which may result in delayed healing and chronic foot ulcers (Jeffcoate and Harding, 2003; Bjarnsholt et al., 2008). Chronic diabetic foot ulcers may subsequently result in lower limb amputation and thus require prompt medical attention.

Precise, objective, reliable, and quantitative measurements of wounds are needed to determine prognosis and ensure best strategies adopted in treating and preventing the development of ulcers. Common clinical assessments for diabetic ulcers include gross observation of wound size, colour, and depth (tendon/capsule/bone involvements), as well as the presence of gangrene, infection, and/or ischaemia (Oyibo et al., 2001; Gul et al., 2006). However, these assessments rely only on the observation of the wound appearance, which are not shown to reflect the underlying histology and the biomechanical strength. During wound healing, collagen, which is a major protein in the cutaneous extracellular matrix, is deposited, organised, and contributing to the biomechanical properties as well as the integrity of the skin. The biomechanical properties can thus be a potential quantitative measurement for chronic wounds such as diabetic ulcers to reflect the functional histological integrity of the wounds. Conventional tensile testing is a common strategy to measure the tensile biomechanical properties which are dependent on the abundance, alignment and orientation of collagen, in particular type I collagen (Silver et al., 1992). Therefore, tensile testing is useful in assessing wound healing and recovery of collagen histology in the literature (Howes et al., 1929; Dogan et al., 2009; Dadpay et al., 2012; Minossi et al., 2014; Lau et al., 2015). Preclinical studies have consistently concluded that impaired wound healing in diabetic condition is characterised by decreased tensile strength, as well as reduced collagen abundance and organisation (Yue et al., 1987; Davidson et al., 1997; Schäffer et al., 1997; Reddy et al., 2001; Immonen et al., 2013; Minossi et al., 2014; Zhang et al., 2016). Nevertheless, both tensile testing and histological examination are not feasible in clinical settings because the excision of tissue samples from the subject is required. Optical coherence tomography (OCT) has been applied to assess *in vivo* skin in real time and non-invasively. Advanced OCT systems were also developed to even image the skin in human at high resolution but only required brief contact (Monnier et al., 2020). Recently, our research team conducted a pilot study utilising an OCT-based air-jet indentation system to assess the indentation stiffness of diabetic wounds in a non-contact manner (Choi et al., 2015). By using the air-jet as an indenter, deformation of the wounds can be achieved by a small force in a non-contact manner, which minimises the risk of contamination or damage due to direct contact. We have demonstrated that the OCT-based air-jet indentation system can accurately and reliably measure the indentation force and deformation in the plantar tissues of patients with diabetes (Chao et al., 2011). Unlike tensile testing that involves the extraction and rupture of specimens, the OCT-based air-jet indentation system reversibly deforms the wounds inward to measure their indentation stiffness at low load and strain (i.e., it does not disrupt the wound tissue). The load of indentation is mainly absorbed by the cutaneous proteoglycans in the extracellular matrix (Silver et al., 1992), which participate in regulating collagen deposition and maturation (Raghow, 1994; Reed and Iozzo, 2002) as well as in the whole wound healing process (Werner and Grose, 2003; Schultz and Wysocki, 2009). Together with our earlier study that demonstrated the negative correlations between the tensile strength and indentation stiffness of a diabetic rat wound model (Choi et al., 2015), we hypothesised that the indentation stiffness is also associated with the recovery of type I collagen histology during wound healing.

The objective of the present study was therefore to establish the correlations between indentation stiffness, and type I collagen abundance and organisation in a diabetic rat wound model.

## Materials and Methods

### Animal Handling and Diabetes Induction

The protocol of this study was approved by the Animal Subjects Ethics Sub-Committee of the Hong Kong Polytechnic University. All the rats received humane care and the protocols were in compliance with the guidelines and regulations from the Animal Subjects Ethics Sub-Committee and Institutional Animal Care and Use Committee^1^. Ten-week-old male (300–400 g) Sprague-Dawley rats used in this study were obtained from the Centralised Animal Facilities of The Hong Kong Polytechnic University. The rats were kept at 21°C and 60% relative humidity under a 12-h light-dark cycle. They were fed with a standard laboratory diet and sterile water *ad libitum*. Before diabetes induction, the rats were fasted for 12 h and their blood glucose level was measured to exclude any abnormally hyper- or hypoglycaemic animals from the study. Intra-peritoneal injection of streptozotocin (50 mg/kg; Sigma-Aldrich, St. Louis, MO, United States) in sterile citrate buffer (pH 4.4) was given to induce diabetes in the rats. After 7 days, the blood glucose level of the rats was measured and monitored once a week throughout the experiment to ensure diabetes was successfully induced and sustained in these rats. Any rats with a blood glucose level lower than 16.7 mM were excluded from the study (King, 2012; Lei et al., 2020).

### Wound Induction

Prior to wound induction, the rats were anaesthetised by an intra-peritoneal injection of a mixture of ketamine (100 mg/kg) and xylazine (3.33 mg/kg). After shaving and cleansing the skin, wounds were induced with a 6 mm biopsy punch on the lateral side of each hind limb (about 3 mm distal to the fibular head). The wounds were left opened without dressing. The rats were then housed individually to prevent cannibalism. At the time points of harvest, photos of the wounds were taken and the wound area was estimated by Fiji software (Schindelin et al., 2012). Percentage of wound area was defined as the area of the wound at a particular time point divided by the initial wound area (post-wounding day 0).

### OCT-Based Air-Jet Indentation Assessment

**FIGURE 1:**
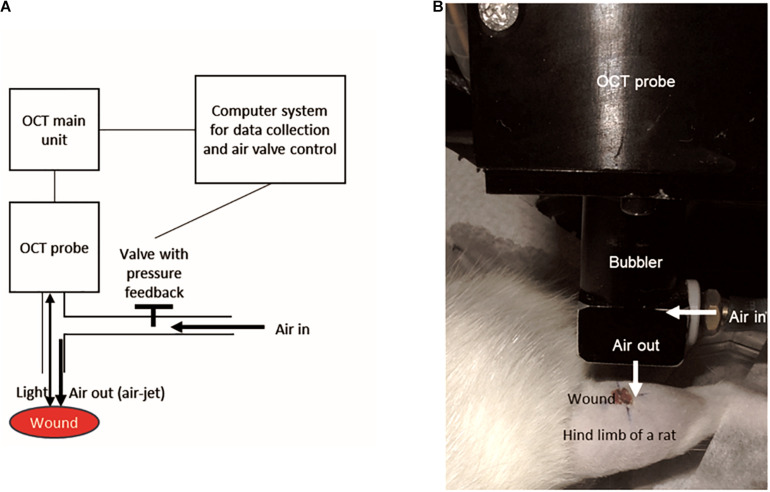
The design of the OCT-based air-jet indentation system used for measuring indentation stiffness of wounds. **(A)** Schematic diagram showing that air-jet whose pressure or flow was controlled by a valve with pressure feedback and a computer system. The air-jet was used as an indenter while the reflected light signal is captured by the OCT probe so that the displacement was detected by the computer system. **(B)** A wound on a hind limb of a diabetic rat was assessed by the OCT-based air-jet indentation system.

Wounds were randomly selected for assessment using the OCT-based air-jet indentation system (Choi et al., 2015) on post-wounding days 3, 5, 7, 10, 14, and 21 so that data from various phases of wound healing could be collected. Immediately before the assessment, the rats were anaesthetised by ketamine and xylazine injection as mentioned. The technical details of the OCT-based air-jet indentation system have been documented in our previous studies (Huang et al., 2009; Choi et al., 2015). The schematic diagram is shown in Figure 1.

Briefly, the probe of the OCT device consisted of a 1-mm air-jet bubbler together with a super-luminescent diode light source (Dense Light, DL-CS3055A, Singapore) operating at a central wavelength of 1,310 nm, a nominal −3 dB spectral bandwidth of 50 nm, and a nominal output power of 5 mW. The OCT unit provided an axial resolution of 18 μm and an imaging depth of approximately 2 to 3 mm in highly scattered materials. An electronic proportional valve with pressure feedback (ITV 1030-311L-Q, SMC Corporation, Tokyo, Japan) at a measurement range of 0.5 MPa was installed. In-house OCT software was used to collect the signals and to control the air valve with a step motor.

The system was used to measure the indentation stiffness of the wounds *in vivo*. There were five measurement sites including one at the centre of the wound with reference to the wound margin, and four at the periphery (proximal, distal, medial, and lateral to the wound with reference to the fibula, 3 mm from the centre of the wound). Two measurements were made at each site with a 5 min resting interval between each measurement; a total of ten measurements were taken for each wound. Three cycles of loading and unloading at an indentation rate of around 0.13 mm/s were applied and recorded, which lasted for approximately 30 s in total. The maximum indentation force was approximately 0.012 N. The deformation was measured by the OCT probe as the inward displacement of the surface of the wound, in accordance with the shift of the OCT signal corresponding to the tissue surface. The stiffness coefficient presented as force/deformation ratio (N/mm) was calculated to represent indentation stiffness. The first loading and unloading cycle was the preconditioning cycle. The indentation stiffness measurements calculated in the loading phases of the second and third cycles were averaged. The typical load-deformation curve including both loading and unloading phases in each cycle is illustrated in Supplementary Figure 1.

### Histological Analysis of Collagen

The full-thickness wounds of randomly selected rats were harvested on post-wounding days 3, 5, 7, 10, 14, and 21 with 8 mm biopsy punches. The wound tissues were then fixed, processed, embedded in paraffin wax, and then sectioned into 5 μm thickness. The sections of the wound centre, which is defined by the sections with the largest wound gap between morphologically mature epidermis and dermis amongst the series of consecutive sections, were deparaffinised and stained with picrosirius red (Sigma-Aldrich, St. Louis, MO, United States) according to the standard procedures (Kiernan, 2002). Type I collagen is the only type of collagen to appear red upon the staining and examination under a polarised light microscope (Nikon Eclipse 80i, Nikon Corporation, Tokyo, Japan) (Kiernan, 2002). Images of the wound centre were captured using a digital camera (Spot Flex 15.2 64 Mp Shifting Pixel, Diagnostic Instruments Inc., Sterling Heights, MI, United States). The quantification of collagen was executed by Fiji software. The red channel of the images was converted to 8-bit colour depth for analysis. The amount of collagen was quantified as area. The percentage of collagen abundance was represented by the amount of collagen normalised by the area of dermis, where collagen is normally present. The intensity of the staining was measured to represent the alignment of collagen fibrils as better aligned collagen fibrils have a higher degree of birefringence and thus generate brighter image under polarised light microscope (Montes and Junqueira, 1991). Greater Feret length indicates greater continuation of a collagen fibre and also reflects greater degrees of orientation and anisotropy (Melis et al., 2002; Noorlander et al., 2002). The average Feret length of the top ten longest collagen fibres of the wound was calculated to represent the orientation of the collagen fibres (Melis et al., 2002; Noorlander et al., 2002). By selecting six representative regions of interest in the dermis, the energy and coherency values from the Fiji plug-in, OrientationJ, were also obtained as parameters of orientation and anisotropy of collagen fibres by structure tensor evaluation (Rezakhaniha et al., 2012). Briefly, the coherency value ranges from 0 to 1, where a coherency value that is close to 1 indicates coherently oriented fibres of the same direction (Fonck et al., 2009); the energy value, consistent to the coherency value, reflects the directionality of the fibres, but is also a function of the area of fibres (Rezakhaniha et al., 2012).

### Statistical Analysis

Intraclass correlation (ICC) [3, 2] analysis was performed to assess the test-retest reliability. Two-way analysis of variance with *post hoc* Tukey’s tests was conducted to examine differences between indentation stiffness measured at the wound centre and periphery over time. Spearman’s Rho was conducted to explore the correlations between the indentation stiffness, and collagen abundance and organisation. The two-sided significance level was set at 0.05. The ICC coefficient was generated by IBM SPSS Statistics for Windows version 21.0 (Armonk, NY, United States: IBM Corp.). The other statistics were performed by GraphPad Prism version 6.00 for Windows (GraphPad Software, La Jolla, CA, United States)^2^. Data were displayed as individual data points and mean ± standard error of mean.

## Results

The diabetic rat model was induced using streptozotocin injection. The streptozotocin injection led to 21.8% mortality. Among the surviving rats, 4.4% did not develop hyperglycaemia.

### Wound Size, Indentation Stiffness, and Type I Collagen Deposition

**FIGURE 2:**
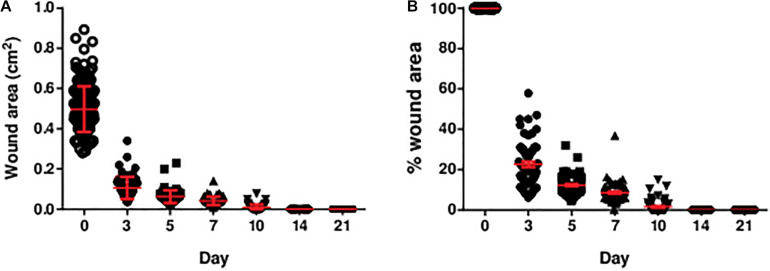
The **(A)** area and **(B)** percentage of area of the diabetic wounds measured over 3 weeks. The area and percentage of area of the diabetic wounds decreased over time. All wounds were closed by post-wounding day 14. The percentage of wound area was the wound area on the specific day divided by the initial wound area (day 0). Data are expressed as individual data points as well as mean ± standard error of mean. Day 0: *n* = 191 wounds, 96 rats; day 3: *n* = 68 wounds, 34 rats; day 5: *n* = 68 wounds, 34 rats; day 7: *n* = 64 wounds, 32 rats; day 10: *n* = 56 wounds, 28 rats; day 14: *n* = 56 wounds, 28 rats; and day 21: *n* = 56 wounds, 28 rats.

Excisional wounds were inflicted on the hind limbs of the rats for the assessment of indentation stiffness, and collagen abundance and organisation. By 14 days, all wounds were grossly closed without notable infection (Figure 2).

Intraclass correlation [3, 2] analysis indicated an excellent test-retest reliability of the indentation stiffness measurement at both the centre and periphery of 46 randomly selected wounds at different time points [ICC (3, 2) = 0.92]. This finding shows that the measurement made in current experimental setting was reliable.

**FIGURE 3:**
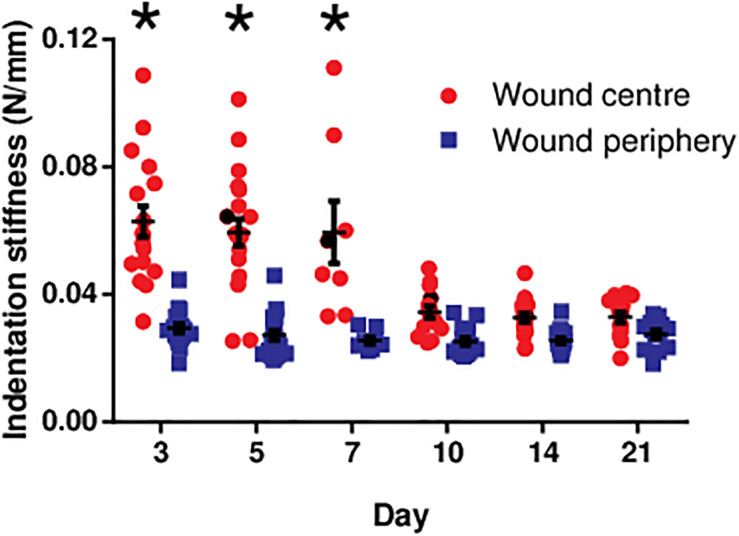
The indentation stiffness measured at the centre and periphery of the diabetic wounds over 3 weeks. The indentation stiffness measurements at the wound centre on days 3, 5, and 7 were significantly greater than those measured on days 10, 14, and 21 (*P < 0.0001). Data are expressed as individual data points as well as mean ± standard error of mean. Day 3: *n* = 17 wounds, 9 rats; day 5: *n* = 20 wounds, 11 rats; day 7: *n* = 8 wounds, 4 rats; day 10: *n* = 14 wounds, 7 rats; day 14: *n* = 16 wounds, 9 rats; and day 21: *n* = 14 wounds, 8 rats.

At the wound centre, the indentation stiffness was comparable in the early phase (days 3, 5, and 7; Figure 3). However, it significantly dropped in the later phase (days 10, 14, and 21; *P* < 0.0001). In contrast, the indentation stiffness at the wound periphery demonstrated negligible change over time and was consistently smaller as compared to the wound centre.

**FIGURE 4:**
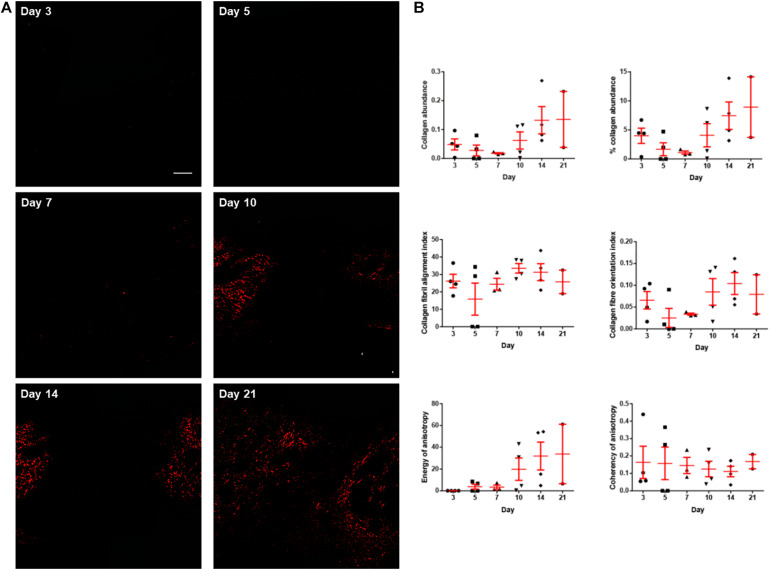
The collagen histology of the diabetic wounds examined over 3 weeks. **(A)** Representative picrosirius red stained sections at the centre of the diabetic wounds examined under polarised light microscope on post-wounding day 3, day 5, day 7, day 10, day 14, and day 21. Type I collagen fibres appear red under polarised light. No collagen was observed to be deposited on days 3 and 5. Very limited collagen was observed on day 7. The abundance of collagen increased as the wounds healed on days 10, 14, and 21. Scale bar = 150 μm. **(B)** The collagen histology including collagen abundance, percentage collagen abundance, collagen fibril alignment, collagen fibre orientation, energy of anisotropy and coherency of anisotropy quantified over 3 weeks. Both abundance of collagen and energy of anisotropy showed obvious increasing trend from post-wounding day 7 to day 21. Data are expressed as individual data points as well as mean ± standard error of mean. Day 3: *n* = 4 wounds, 4 rats; day 5: *n* = 4 wounds, 4 rats; day 7: *n* = 3 wounds, 3 rats; day 10: *n* = 4 wounds, 4 rats; day 14: *n* = 4 wounds, 4 rats; and day 21: *n* = 2 wounds, 2 rats.

Picrosirius red staining was performed to visualise type I collagen fibres (appear in red under polarised light), which is the main contributor to the tensile strength of the wounds and the skin. Images taken at the centre of the wounds harvested on post-wounding days 3, 5, 7, 10, 14, and 21 revealed that the majority of the staining appeared red indicating type I collagen fibres. However, green signals, which indicates thinner and less aligned collagen fibres, such as type III, were negligible in abundance. Type I collagen fibres were quantified in terms of collagen abundance, fibril alignment and fibre orientation (Figure 4). The collagen abundance showed an notable increase from day 7 to day 21. However, there were no substantial changes in collagen organisation (i.e., fibril alignment, fibre orientation, and anisotropy) throughout the study period.

### Correlations Between Indentation Stiffness, and Type I Collagen Abundance and Organisation

**FIGURE 5:**
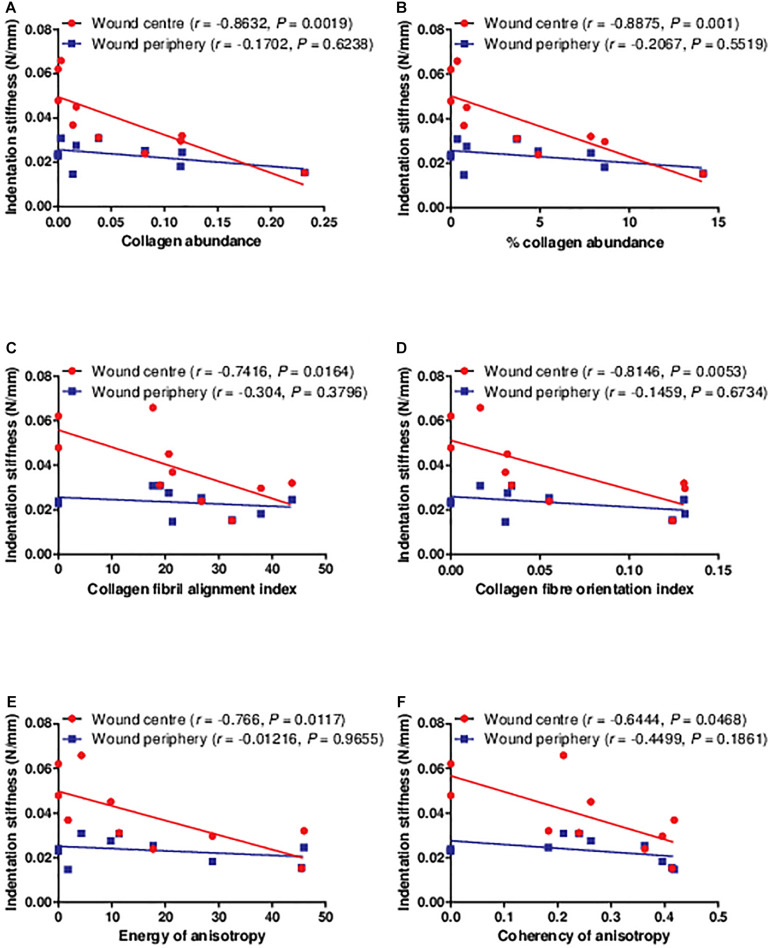
The correlations between the indentation stiffness and collagen histology examined in the diabetic wounds. The indentation stiffness measured at the wound centre was significantly negatively correlated to the **(A,B)** collagen abundance, **(C)** alignment, **(D)** orientation, and **(E,F)** anisotropy on post-wounding day 3 (*n* = 1 wound, 1 rat), 5 (*n* = 2 wounds, 2 rats), 7 (*n* = 2 wounds, 2 rats), 10 (*n* = 1 wound, 1 rat), 14 (*n* = 2 wounds, 2 rats), and 21 (*n* = 2 wounds, 2 rats). Data at different time points were pooled together.

As type I collagen is the major structural protein in the extracellular matrix underlying biomechanical properties, correlations were established between the indentation stiffness and the abundance and organisation of type I collagen. The indentation stiffness at the wound centre was significantly negatively correlated with the collagen abundance (absolute abundance and percentage abundance) and organisation (fibril alignment, fibre orientation, energy of anisotropy, and coherency of anisotropy; Figure 5). As a negative control, the indentation stiffness at the wound periphery was not correlated with the collagen abundance and organisation at the wound centre. These findings confirm that the correlations are region specific (i.e., only indentation stiffness measured at the wound centre, but not periphery, reflects the collagen at the wound centre). Interestingly, such negative correlations between the indentation stiffness at the wound centre (but not the wound periphery) and collagen abundance and organisation were also found significant in non-diabetic wounds of strain-, sex-, and age-matched rats (Supplementary Figure 2).

## Discussion

This study is the first one to show that the abundance and organisation of collagen in wounds could be reflected by an *in vivo*, non-contact and non-invasive biomechanical measurement. This provides further evidence to support the use of indentation stiffness as a biomechanical assessment of diabetic wounds. As our previous study have shown that the indentation stiffness could reflect the tensile properties of diabetic wounds, we postulated that collagen fibril alignment and fibre orientation should also be factors in the correlation (Choi et al., 2015). The current study demonstrating negative correlations between indentation stiffness, and type I collagen abundance and organisation in diabetic wounds may therefore further explain the negative correlations between the indentation stiffness and tensile properties, which are mainly contributed by type I collagen fibres.

Proteoglycans may absorb compressive stress and resist tissue deformation upon compression, thus contributing to the compressive/indenting property of the skin (Silver et al., 1992). Correlation between indentation stiffness and collagen shown in the current study suggests interesting relationships between indenting property, proteoglycans and collagen in wound healing. Proteoglycans, which is a component of the ground substance, have roles in collagen deposition and maturation. Decorin, regarded as one of the small-sized proteoglycans found in the skin, interacts with various types of collagen (Vogel et al., 1984; Brown and Vogel, 1989; Tenni et al., 2002). It plays an important role in regulating collagen deposition (Reed and Iozzo, 2002) and also the recovery of collagen organisation upon injury (Dunkman et al., 2014). Hence, it potentially affects the tensile properties of the skin wounds (Carrino et al., 2000). It should be noted that the abundance of decorin increases following wound closure (Yeo et al., 1991). In contrast, other proteoglycans such as chondroitin sulphate proteoglycans of large molecular size remain dominant during the wound healing process, when the wound is not completely closed (Yeo et al., 1991). Proteoglycans of large size (e.g., chondroitin sulphate proteoglycans) are interlaced with thin collagen fibrils, vice versa, the smaller-sized (e.g., decorin) are distributed among thicker collagen (Kuwaba et al., 2002). Owing to such characteristic, we speculate that the dynamic changes in molecular size of proteoglycans do not only contribute to the organisation and maturation of collagen fibres, but also the indentation stiffness during wound healing. The predominance of large-sized proteoglycans may contribute to an increased indentation stiffness in the early phase (day 3 to day 7), prior to wound closure. They are then replaced by the smaller-sized proteoglycans following wound closure, which may account for the reduced indentation stiffness in the later phase (day 10 to day 21). This may not only justify the negative correlations between the indentation stiffness and collagen abundance and organisation found in the current study, but also explain the consistently smaller indentation stiffness at the wound periphery, which is presumably uninjured skin, with proteoglycans of smaller size (Kuwaba et al., 2002). Future study is warranted to confirm the relationships between the indentation stiffness, proteoglycans, and collagen deposition and organisation of diabetic wounds, in order to provide concrete support on the clinical use of the indentation stiffness measurement.

Although the present study focuses on the dermal layer of the skin/wound, wound healing does involve re-epithelialisation. Particularly in the early phase where the dermal layer is still very thin or even absent, the epidermal layer may probably be the main structure contributing to the biomechanical properties of the wound. Indeed, our data show that the thicker epidermal layer in the early phase (Supplementary Figure 3) coincides with the greater indentation stiffness in the early phase of diabetic wound healing (days 3, 5, and 7; Figure 3). The epidermal thickness at different time points has a significant positive correlation with the indentation stiffness at the wound centre (Supplementary Figure 3). Keratinocyte is the major cell type in the epidermal layer, which synthesises protein fibre keratin that can potentially contribute to compressive/indenting biomechanical properties (Wang et al., 2016). The presence of epidermal layer in the early phase of wound centre in the current study suggests the presence of keratin and keratinocytes at the wound centre in the early phase. Other studies conducted with excisional wounds in rats (Sabol et al., 2012) and humans (Usui et al., 2005) have also found the presence of keratin and keratinocytes in the early phase of wound healing. During wound healing, keratinocytes proliferate, migrate, differentiate into different stages and express distinct types of keratins such as K16 in activated proliferating keratinocytes and K10 in terminally differentiated keratinocytes (Freedberg et al., 2001; Santos et al., 2002; Usui et al., 2008). It has been suggested that different types of keratins with different structures and molecular weights could have different biomechanical properties (Bragulla and Homberger, 2009). Therefore, in different phases of wound healing, different compositions and abundance of various types of keratins may possibly contribute to the different indentation stiffness. This may also explain why the indentation stiffness at the wound centre is significantly different from that at the periphery as the compositions of keratins at these sites are probably different. At the wound centre, it is expected that keratinocytes are more activated and proliferative expressing high level of K16. Conversely, keratinocytes in the uninjured area at the wound periphery express less K16 but more K10. Interestingly, keratinocytes expressing different types of keratins release different signalling molecules such as cytokines (e.g., TNF-α) and growth factors (TGF-β) to regulate the signalling and differentiation of fibroblasts. The release of various signalling molecules has potential effects on the collagen deposition and maturation in dermis (Werner et al., 2007; Pastar et al., 2014). Future studies are needed to clarify the indentation stiffness of specific types, compositions and abundance of keratins in *in vivo* wound models and, in addition, to examine the regulation of collagen deposition and maturation in dermal layer by keratinocytes expressing distinct types of keratins in wound healing process. This series of studies will hopefully be able to further elucidate the biomechanical and molecular characteristics of the wounds reflected by the indentation stiffness in different phases of wound healing.

Apart from the possible contribution by proteoglycans and keratins, the consistently high indentation stiffness in the early phase of wound healing could be related to wound oedema. The histological finding in the present study suggests that oedema was present in the wounds on days 3, 5, and 7 but not on days 10, 14, and 21 (Supplementary Figure 3). This observation is consistent with the report by Vexler et al. (1999) who reported skin oedema to be associated with increased stiffness. In addition, it is generally agreed that oedema increases the stiffness of the tissue due to hydrostatic pressure in a confined space. The hypothesis that the high indentation stiffness is related to wound oedema is also partly supported by the present finding that the indentation stiffness at the wound periphery, where no wound oedema was found, was substantially smaller than that at the wound centre.

The main focus of the current study is to evaluate the correlations between the indentation stiffness, and type I collagen abundance and organisation in diabetic wounds. Nevertheless, to the best of our knowledge, this study is the first study to illustrate the quantitative changes in collagen abundance and organisation of diabetic wounds, over a period of time from the early phase (post-wounding day 3) to the later phase (day 21). Existing literature on the collagen histology is lacking the presentation of changes at various time points. Therefore, the present findings may provide insights to the research on optimal time points for specific interventions to diabetic wounds in different phases of wound healing. For the collagen abundance, our data show a non-significant decreasing trend from post-wounding day 3 to day 7 and an increasing trend thereafter. Majority of the studies which investigated the time course changes in collagen using either diabetic (Ponrasu and Suguna, 2012; Ram et al., 2015; Zhang et al., 2016) or non-diabetic wound models (Oxlund et al., 1996; Ponrasu and Suguna, 2012; Almeida et al., 2014) demonstrated an increase in collagen abundance over time and plateau or slightly decreases in the very late phase, which is consistent with the rising trend from day 7 to day 21 concluded in the current study. Therefore, the non-significant decrease from day 3 to day 7 in the collagen abundance shown in the present study could be attributed to individual variation. However, since limited study has focussed on the time course changes of collagen abundance between day 3, or earlier, and day 7 in diabetic wound models, we are unable to rule out the possibility that the decreasing trend of collagen abundance on post-wounding day 7 is as a result of diabetes-related complications in wound healing.

For collagen fibril alignment and fibre orientation indices, as well as the coherency of anisotropy which is also a measurement of collagen fibre orientation estimated by tensor analysis, there was no obvious increase or decrease across the time points. On the other hand, the energy of anisotropy is a function of both area and orientation of fibres. The rising trend of the energy of anisotropy corresponds to the increasing trend of the collagen abundance. In agreement with the unchanged collagen fibril alignment and fibre orientation in the current study, our previous study have also found no significant difference in the collagen fibril alignment and fibre orientation between post-wounding day 7 and day 14 in a diabetic rat excisional wound model (Choi et al., 2016). Broadley et al. (1989) demonstrated that even though the collagen concentration in diabetic wounds approached to the non-diabetic wound level over time, the tensile strength of the diabetic wounds remained impaired. Since tensile strength is contributed by all collagen abundance, alignment and orientation, they extrapolated that wounds in diabetic rat model might have deficits in the recovery of collagen alignment and orientation (Broadley et al., 1989). To date, no other published studies have quantitatively assessed the collagen fibril alignment and fibre orientation in wounds over a period of time. We therefore believe that the recovery of collagen alignment and orientation may not be significant in the current diabetic wound model within the experimental period, which is in agreement with the literature.

In conclusion, we established correlations between the indentation stiffness, type I collagen abundance and organisation of diabetic wounds in a rat model. Our findings provide evidence supporting the potential clinical use of the OCT-based air-jet indentation system for biomechanical assessment of diabetic wounds. In the future, we anticipate investigations using a more clinically relevant wound model involving repetitive stress and chronic inflammation resembling foot ulcers in patients to explore the correlations between the indentation stiffness and more clinically oriented parameters such as the prognosis of the wounds, risk of infections and severity of diabetes.

## Data Availability Statement

The raw data supporting the conclusions of this article will be made available by the authors, without undue reservation.

## Ethics Statement

The animal study was reviewed and approved by the Animal Subjects Ethics Sub-Committee of the Hong Kong Polytechnic University.

## Author Contributions

HC, AC, and GC: conceptualisation. HC: data curation, formal analysis, visualisation, and writing – original draft. GC: funding acquisition and project administration. HC and MN: investigation. YZ: methodology. AC and GC: supervision. HC, AC, YZ, Y-KJ, and GC: writing – review and editing. All authors contributed to the article and approved the submitted version.

## Conflict of Interest

The authors declare that the research was conducted in the absence of any commercial or financial relationships that could be construed as a potential conflict of interest.

## Footnotes

1 http://www.polyu.edu.hk/ro/forms/Attachment%202_Guidelines%20for%20the%20assessment%20&%20mgmt%20of%20pain.pdf

2 www.graphpad.com
