# Supplementary material for: Indentation Stiffness Measurement by an Optical Coherence Tomography-Based Air-Jet Indentation System Can Reflect Type I Collagen Abundance and Organisation in Diabetic Wounds
Source: Front Bioeng Biotechnol. 2021 Mar 4;9:648453. doi: 10.3389/fbioe.2021.648453 (PMC7969662; doi:10.3389/fbioe.2021.648453)
Supplement: Supplementary file 1 [file Image_1.pdf]

Supplementary Material

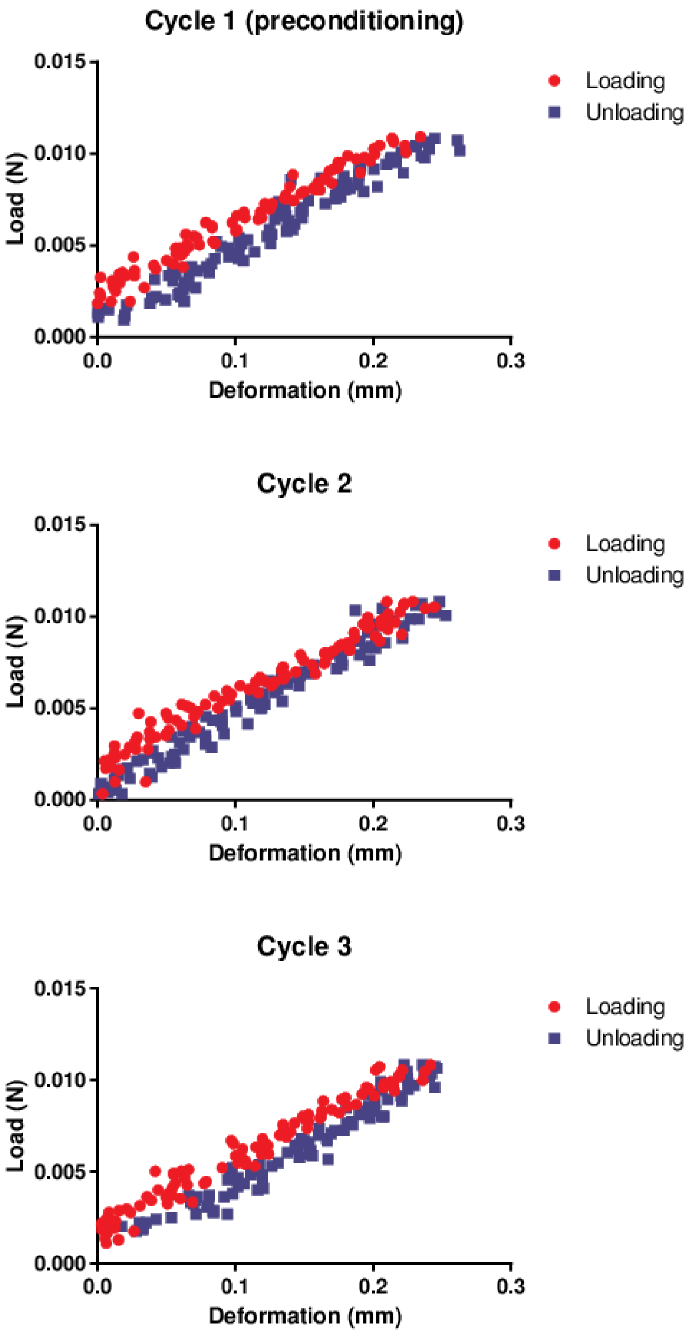

Supplementary Figure 1. Typical load-deformation curves obtained from a wound on post-wounding day 21 which was assessed by the OCT-based air-jet indentation system. This system applied air-jet to deform/indent the wound for three loading and unloading cycles. Indentation stiffness was derived as the average of the slope of the curves from the second and third cycles.

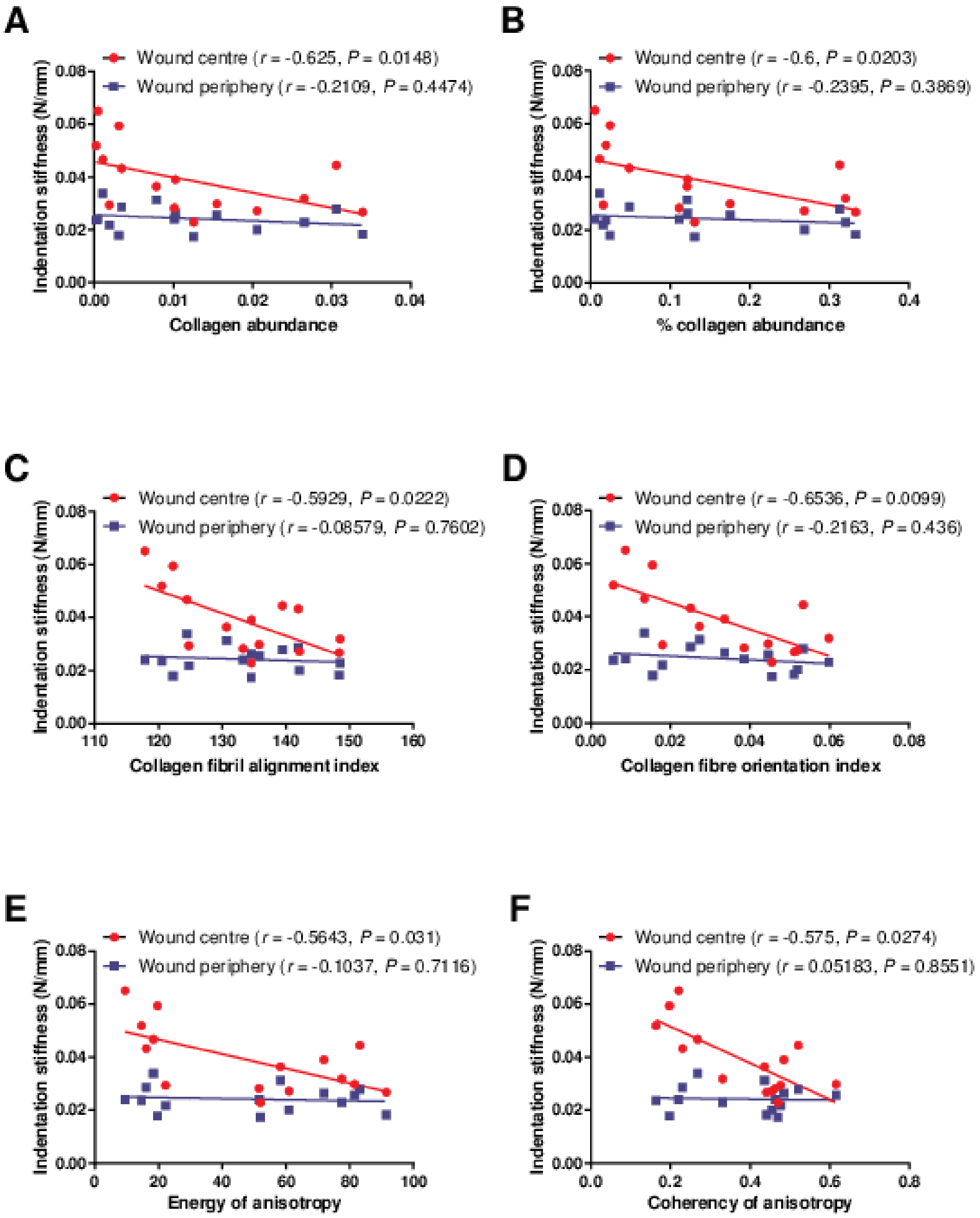

Supplementary Figure 2. The correlations between the indentation stiffness and collagen histology examined in the non-diabetic wounds. The indentation stiffness measured at the wound centre was significantly negatively correlated to the (A, B) collagen abundance, (C) alignment, (D) orientation and (E, F) anisotropy on post-wounding day 3 (n = 2 wounds, 2 rats), 7 (n = 3 wounds, 3 rats), 10 (n = 3 wounds, 3 rats), 14 (n = 2 wounds, 2 rats) and 21 (n = 5 wounds, 5 rats). Data at different time points were pooled together.

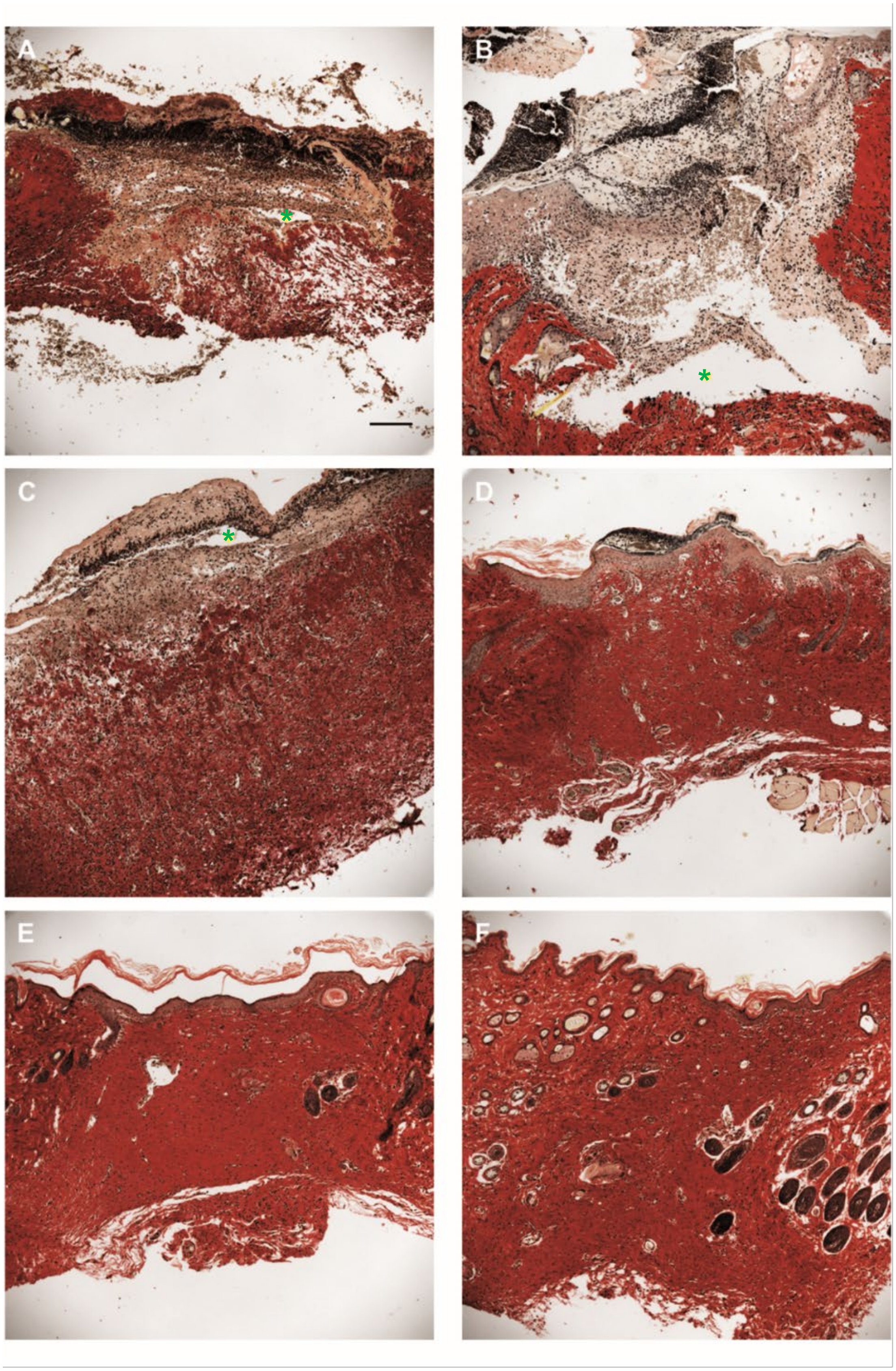
A
B
C
D
E
F

G

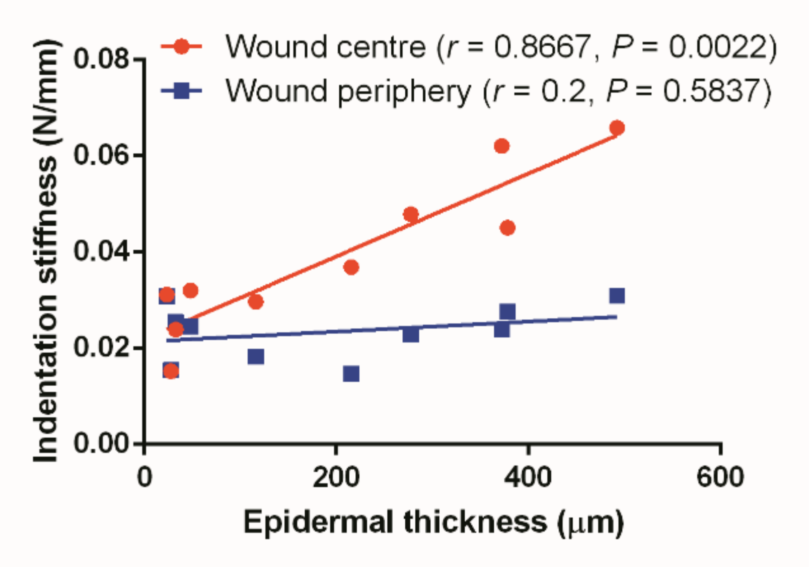

Supplementary Figure 3. Representative picrosirius red stained sections at the centre of the diabetic wounds examined in bright field on post-wounding (A) day 3, (B) day 5, (C) day 7, (D) day 10, (E) day 14 and (F) day 21. The thickness of epidermal layer decreased gradually from day 3 to day 21. (G) Interestingly, the epidermal thickness measured at the wound centre was significantly correlated with the indentation stiffness at the wound centre. The apparently empty space in the sections (marked by green asterisks) suggests the presence of oedema. Possible oedema was found in the wounds on days 3, 5 and 7 but not on days 10, 14 and 21. Scale bar = 150 µm.
